# Impact of plastic-related compounds on the gene expression signature of HepG2 cells transfected with CYP3A4

**DOI:** 10.1007/s00204-023-03648-4

**Published:** 2023-12-30

**Authors:** Matteo Rosellini, Ejlal A. Omer, Alicia Schulze, Nadeen T. Ali, Joelle C. Boulos, Federico Marini, Jan-Heiner Küpper, Thomas Efferth

**Affiliations:** 1 Department of Pharmaceutical Biology, Institute of Pharmaceutical and Biomedical Sciences, Johannes Gutenberg University, Staudinger Weg 5, 55128 Mainz, Germany; 2 Institute of Medical Biostatistics, Epidemiology and Informatics (IMBEI), Medical Center of the Johannes Gutenberg University, 55122 Mainz, Germany; 3 Research Center for Immunotherapy (FZI), Langenbeckstraße 1, 55131 Mainz, Germany; 4 Institute of Biotechnology, Brandenburg University of Technology Cottbus-Senftenberg, 03046 Senftenberg, Germany

**Keywords:** Cytotoxicity, Ecotoxicity, Environmental pollution, Hepatotoxicity, Marine pollution, Microplastic, RNA-sequencing

## Abstract

The presence of plastic and microplastic within the oceans as well as in marine flora and fauna have caused a multitude of problems that have been the topic of numerous investigations for many years. However, their impact on human health remains largely unknown. Such plastic and microplastic particles have been detected in blood and placenta, underlining their ability to enter the human body. Plastics also contain other compounds, such as plasticizers, antioxidants, or dyes, whose impact on human health is currently being studied. Critical enzymes within the metabolism of endogenous molecules, especially of xenobiotics, are the cytochrome P450 monooxygenases (CYPs). Although their importance in maintaining cellular balance has been confirmed, their interactions with plastics and related products are poorly understood. In this study, the possible relationship between different plastic-related compounds and CYP3A4 as one of the most important CYPs was analyzed using hepatic cells overexpressing this enzyme. Beginning with virtual compound screening and molecular docking of more than 1000 plastic-related compounds, several candidates were identified to interact with CYP3A4. In a second step, RNA-sequencing was used to study in detail the transcriptome-wide gene expression levels affected by the selected compounds. Three candidate molecules ((2,2′-methylenebis(6-*tert*-butyl-4-methylphenol), 1,1-bis(3,5-di-*tert*-butyl-2-hydroxyphenyl)ethane, and 2,2′-methylenebis(6-cyclohexyl-4-methylphenol)) had an excellent binding affinity to CYP3A4 in-silico as well as cytotoxic effects and interactions with several metabolic pathways in-vitro. We identified common pathways influenced by all three selected plastic-related compounds. In particular, the suppression of pathways related to mitosis and ‘DNA-templated DNA replication’ which were confirmed by cell cycle analysis and single-cell gel electrophoresis. Furthermore, several mis-regulated metabolic and inflammation-related pathways were identified, suggesting the induction of hepatotoxicity at different levels. These findings imply that these compounds may cause liver problems subsequently affecting the entire organism.

**Supplementary Information:**

The online version contains supplementary material available at 10.1007/s00204-023-03648-4.

## Introduction

During the past 30 years, the global plastic use has quadrupled (Global Plastics Outlook 2022). The reason is simple: due to its low-cost, flexibility, strength, and ease of manufacturing, it facilitates our everyday life. However, in the face of its advantageous versatility, the use of plastic entails enormous negative consequences. Plastic remains in the environment for more than 100 years (Jambeck et al. 2018) and decomposes into microplastic (< 5 mm) or nanoplastic (< 0.1–1 μm) particles (Koelmans et al. 2022). Thereby, plastic pollution has caused massive problems to the oceans (Cózar et al. 2014; Eriksen et al. 2014; Vince and Hardesty 2016; Efferth and Paul 2017), to marine animals (Alomar et al. 2017; Anbumani and Kakkar 2018; de Sá et al. 2018) and to the environment in general (da Costa et al. 2016; Prata 2018). However, little is known about the impact on human health. Microplastic may be ingested through the consumption of food and water (Danopoulos et al. 2020; Oliveri Conti et al. 2020) and can pass the gastrointestinal epithelial barrier (Wright and Kelly 2017). Through respiration, it can also be detected in the lungs (Amato-Lourenço et al. 2021). This is underlined by recent findings of microplastic in blood (Leslie et al. 2022), placenta (Ragusa et al. 2021), and breast milk (Ragusa et al. 2022). During the production of plastic, plasticizers, flame retardants, dyes, or antioxidants can be utilized. If released, these plastic-related compounds can also interact with human cells (Koch and Calafat 2009; Meeker et al. 2009). Despite the enormous presence of plastic, microplastic, and related compounds in the environment, their consequences remain undetermined once inside the human body. Being xenobiotic, plastic-related compounds may interact with enzymes that play a leading role in the biotransformation of a large range of endogenous as well as exogenous compounds (Guengerich 2008; Esteves et al. 2021). Such enzymes are the cytochrome P450 monooxygenases (CYP), which are highly expressed in hepatocytes (Sevior et al. 2012). One of the most important CYPs is CYP3A4. It displays broad substrate specificity and interacts with numerous endogenous compounds including testosterone (Li et al. 1995), therapeutic drugs such as clonazepam, and also pollutants that influence its activity (Zhuang et al. 2017; Chen and Liu 2019). Previously, the interaction of chemical pollutants with cytochromes has been discovered (Chen et al. 2019). However, the possible relationship between plastic including all its related compounds and CYP3A4 remains unknown, despite being increasingly hazardous for human health (The Lancet Planetary Health 2017; Jiang et al. 2020). In this study, we investigated the effects of compounds used for plastic production on CYP3A4 by using CYP3A4-transfected HepG2 cells. Initially, we identified compounds interacting with CYP3A4 from a large library of plastic-related chemicals using in-silico screening. Then, based on RNA-sequencing, the transcriptome-wide gene expression affected by the selected compounds on CYP3A4-overexpressing HepG2 cells was determined. These results, which were corroborated by cell cycle and single cell gel electrophoresis analyses, permitted an estimation on the scope of which these compounds may influence human health in general and specifically hepatotoxicity.

## Materials and methods

### Chemicals

Compound **1**: dicyclohexyl phthalate (CAS 84-61-7, > 99%), compound **2**: diisobutyl phthalate (CAS 84-69-5, > 98%), compound **3**: octrizole (CAS 3147-75-9, > 98%), compound **4**: 2,2′-methylene bis(6-*tert*-butyl-4-methylphenol) (CAS 119-47-1, > 99%), compound **6**: 2,2′-methylene bis(6-cyclohexyl-4-methylphenol) (CAS 4066-02-8, > 97%). The compounds were acquired from TCI Deutschland GmbH (Eschborn, Germany). Compound **5**: 1,1-bis(3,5-di-*tert*-butyl-2-hydroxyphenyl)ethane (CAS 35958-30-6, 96%) was acquired from abcr GmbH, Karlsruhe, Germany.

### Cell lines

CYP3A4-overexpressing HepG2 cells were established as previously described (Herzog et al. 2015). Cells were grown in DMEM medium (DMEM, 31966021, Gibco™) at 37 °C and 5% CO2 in a humidified incubator. DMEM media were supplemented with 10% fetal bovine serum (FBS) and with 3 μg/mL blasticidin (ant-bl-05, InvivoGen).

### PyRx screening

PyRx (https://pyrx.sourceforge.io) was used for preliminary screening of the binding affinities of the test compounds to CYP3A4. We screened more than 1000 compounds associated with plastic production whose three-dimensional ligand structures were downloaded as standard data files from PubChem (NCBI, MD, USA) (Kim et al. 2021). The crystal structure of CYP3A4 was downloaded from the Protein Data Bank (http://www.rcsb.org/) (Berman 2008) as a PDB file (PDB code: 3NXU) (Sevrioukova and Poulos 2010).

### Molecular docking

Molecular docking was performed for the compounds with the lowest PyRx binding energies. The binding affinities of the top 70 compounds were calculated using AutoDock 4.2. A grid box was positioned around the drug-binding sites of CYP3A4 with the center of the grid box at *x* = 31.872, *y* = −21.814, and *z* = 28.756 and with the number of grid points (npts) being 80 in *x*, 90 in *y*, and 80 in *z*. Molecular docking was executed with the Lamarckian Genetic Algorithm, with 250 runs and 25 million evaluations. Discovery Studio Visualizer software was used for visualizing protein–ligand interactions. Part of the research was conducted using the supercomputer Mogon II and advisory services offered by Johannes Gutenberg University Mainz (hpc.uni-mainz.de), which is a member of the AHRP (Alliance for High-Performance Computing in Rhineland Palatinate, www.ahrp.info) and the Gauss Alliance e.V.

### Cytotoxicity assay

The cytotoxicity of six compounds selected from in-silico screening was analyzed through a resazurin assay. Aliquots of 10^4^ CYP3A4-overexpressing HepG2 cells were seeded per well into 96-well plates. Cells were treated with different concentrations of the selected compounds ranging from 0.003 to 100 µM in a total volume of 200 µL for 72 h. Then, 20 µL/well of resazurin 0.01% w/v (Sigma Aldrich, Taufkirchen, Germany) was added. The fluorescence intensity was measured with the Infinite M200 Pro-plate reader (Tecan, Crailsheim, Germany). Dose–response curves were generated from three independent experiments for each compound and 50% inhibition concentrations (IC_50_) were calculated. The analysis was represented using Prism 6 GraphPad Software (La Jolla, CA, USA).

### RNA extraction

Aliquots of 5 × 10^5^ CYP3A4-overexpressing HepG2 cells were seeded into six-well-plates 24 h before treatment. Cells were treated with previously calculated IC_50_ concentrations of the compounds of interest. Control (ctrl) cells were treated with 0.2% DMSO. After 24 h incubation, the cells were harvested. RNA extraction was performed with the InviTrap®Spin Cell RNA Mini Kit (Invitek Molecular GmbH, Berlin, Germany), according to the manufacturer's instruction. Briefly, the cell pellet was lysed with 350 μL Lysis Solution and treated with β-mercaptoethanol. After DNA removal, 350 μL 70% ethanol was added and the sample was applied to the RNA-RTA Spin Filter. After several washing steps, RNA was eluted with 60 μL of Elution Buffer R and the concentration and purity measured with NanoDrop.

### RNA-sequencing

Next-generation sequencing was conducted by StarSEQ GmbH, Mainz, Germany. The quality of the extracted RNA was verified by the company with a 2100 Bioanalyzer system (Agilent Technologies, CA, USA). After mRNA isolation and library preparation using the NEBNext^©^ Ultra™ II Directional RNA Library Prep Kit (New England Biolabs, MA, USA), RNA sequencing of around 25 mio PE reads (2 × 12.5 M reads, 2 × 150 nt) was performed with the Illumina NextSeq 2000™ system. Each treatment and control group included two replicates.

### Bioinformatics analysis

Quality control on the sequencing data was performed with the FastQC tool (0.12.0, https://www.bioinformatics.babraham.ac.uk/projects/fastqc/). Transcript abundance estimates were computed with Salmon (version 1.5.0) (Patro et al. 2017) with a transcriptome index generated from the GENCODE (version 38), and subsequently summarized to gene level with the tximeta R package (version 1.16.0) (Love et al. 2020). The exploration, modelling, and interpretation of the expression data followed previously described protocols (Ludt et al. 2022). Exploratory data analysis was executed with the pcaExplorer package (version 2.24.0) (Marini and Binder 2019). Differential expression analysis was performed with DESeq2 package (version 1.38.3) (Love et al. 2014), setting the false discovery rate (FDR) cutoff to 0.05. Accurate estimation of the effect sizes (described as log2 fold change) was finalized using the apeglm shrinkage estimator (version 1.20.0) (Zitovsky and Love 2020). Subsequent analyses included Gene Ontology pathway enrichment by topGO (version 2.50.0) (Alexa et al. 2006)—using all expressed genes as background dataset and the ideal package (version 1.22.0) (Ludt et al. 2022)—and by clusterProfiler (version 4.6.0) (Wu et al. 2021) with default settings using the log fold change as input. The enrichment results were the foundation for visualization and summarization with the GeneTonic package (version 2.2.0) (Marini et al. 2021). Gene expression profiles were plotted as heatmaps (color-coded standardized *Z* scores for the expression values, after variance stabilizing transformation) to simplify comparison across samples.

### Cell cycle analysis

CYP3A4-overexpressing HepG2 cells were treated with IC_50_ concentration of compounds **4**, **5** and **6** and DMSO (ctrl). After 24 h of incubation, cells were fixed with ethanol and stored at −20 °C overnight. Then, samples were centrifuged and resuspended in 1 mL cold PBS containing 1 mg/mL RNase (Sigma-Aldrich, Taufkirchen, Germany) and 50 μg/mL PI (Sigma-Aldrich, Taufkirchen, Germany). The measurement was performed using a BD LSRFortessa™ Cell Analyzer (Becton–Dickinson, Heidelberg, Germany). The results were analyzed using FlowJo software (Celeza, Olten, Switzerland).

### Single-cell gel electrophoresis

The OxiselectTM Comet Assay Kit (3-Well Slides) (Cell Biolabs/Biocat, Heidelberg, Germany) was used to perform the comet assay. CYP3A4-overexpressing HepG2 cells were seeded in 6-well plates and treated with compounds **4**, **5**, and **6** at their respective IC_50_ concentrations, along with DMSO (negative control), for 24 h. H_2_O_2_ (50 µM) was used as a positive control and added to the cells for 1 h. After harvesting, the cells were centrifuged and resuspended in cold PBS. Then, the cell suspension was mixed with molten agarose at 37 °C. Subsequently, the samples were spread on comet slides and dried. Pre-cooled lysis buffer and pre-cooled alkaline electrophoresis solution buffer were applied to the slides in the darkroom at 4 °C. The slides were then placed horizontally in the alkaline electrophoresis solution buffer in the electrophoresis chamber. The chamber was subjected to a voltage of 25 V for 20 min. Afterward, the slides were washed with distilled water, followed by cold 70% ethanol, and dried overnight. Vista Green DNA dye was added to the slides (100 µL per well). DNA damage was observed using the EVOS digital inverted microscope (Life Technologies GmbH, Darmstadt, Germany). Sixty comets for each treatment were randomly selected and analyzed using the OpenComet plugin in Image J software (National Institutes of Health). The Tail DNA% was measured as a parameter for DNA damage (Gyori et al. 2014).

### Predicted metabolites

SmartCYP (Rydberg et al. 2010a, b) was used to predict the possible metabolism sites of the three compounds. Successively, we used ChemDraw software to modify the initial molecules by adding the hydroxyl group to each of the first three predicted ranking sites and in different combinations. Then, the binding affinities of the metabolite compounds were calculated using AutoDock 4.2. The grid was placed on the CYP3A4 drug binding sites with the grid center at *x* = 31.872, *y* = −21.814 and *z* = 28.756 and with a number of grid points (npts) of 80 in *x*, 90 in *y* and 80 in *z*. Molecular docking was performed using the Lamarckian genetic algorithm, with 250 runs and 25 million evaluations. Finally, the analysis was represented using Prism 6 GraphPad software (La Jolla, CA, USA).

## Results

### PyRx screening

**Fig. 1:**
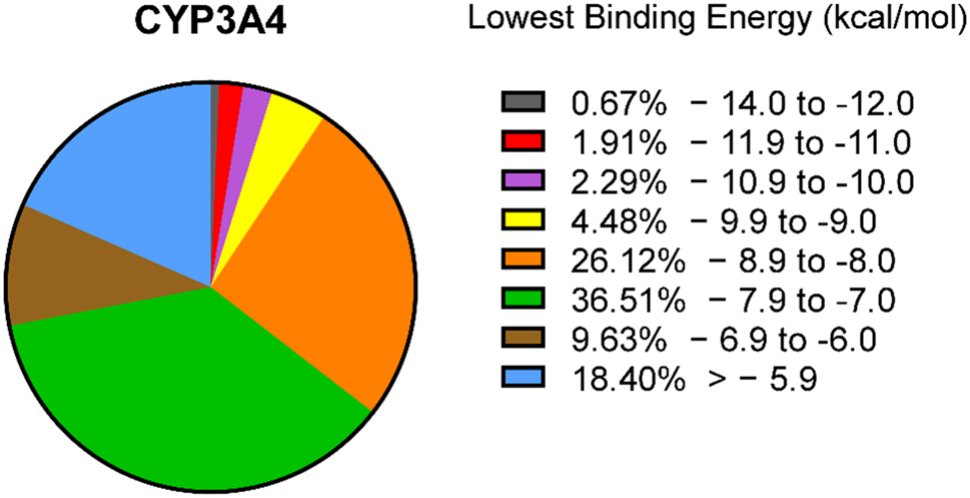
Virtual drug screening using PyRx. The pie chart illustrates the percentage of compounds within a specific range of lowest binding energy of CYP3A4

Using the PyRx software, we screened more than 1000 plastic-associated compounds assembled from PubChem. As shown in Fig. 1, the majority of compounds has a binding affinity on CYP3A4 between −7.9 and −7.0 kcal/mol. A small fraction (8%) of the selected compounds were in a range from −9.0 to −14.0 kcal/mol, indicating a high binding affinity to CYP3A4. We selected the best 70 compounds for subsequent analyses.

### Molecular docking

**Fig. 2:**
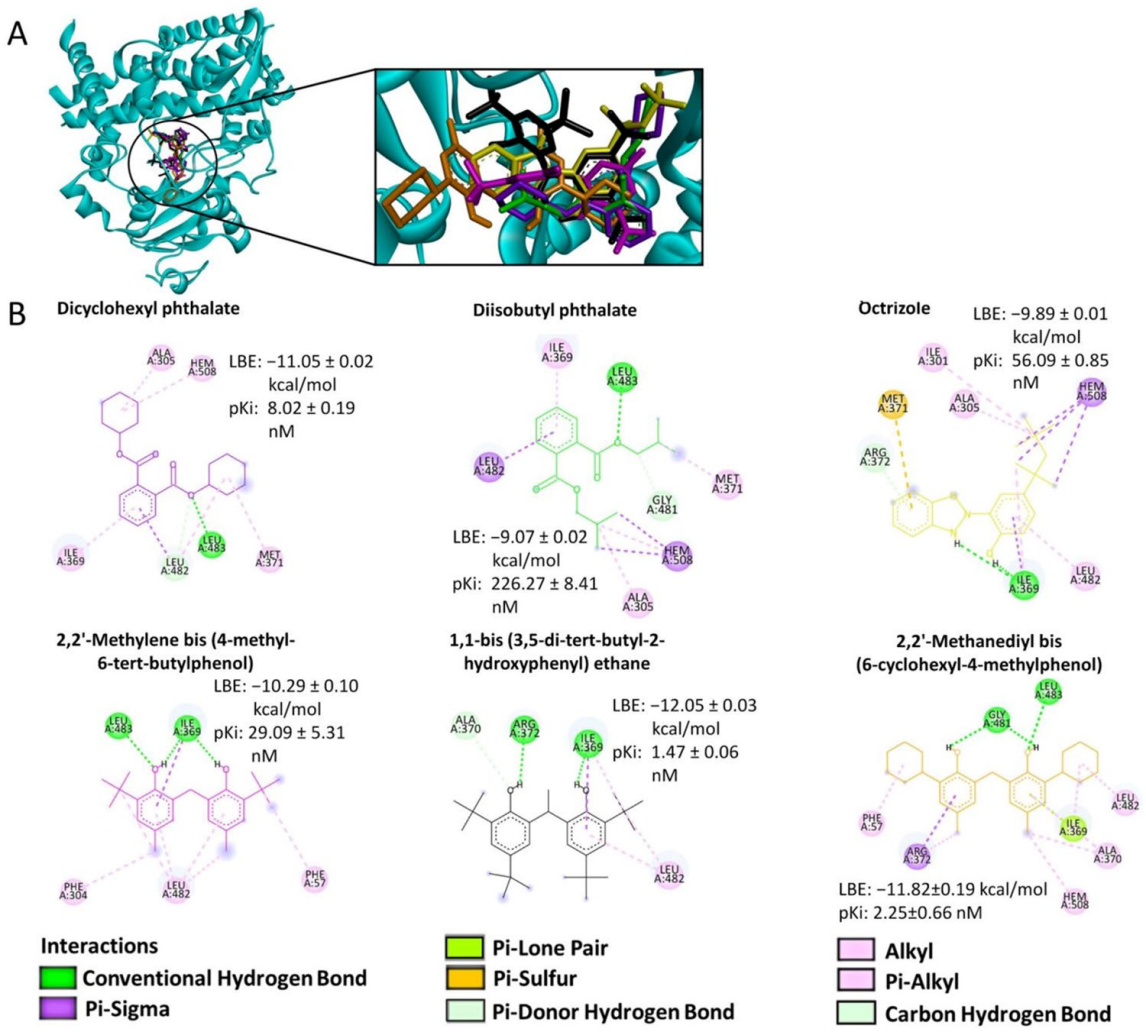
Representation of the binding mode between six selected compounds and CYP3A4. **A** 3D structure of a cytochrome 3A4 (cyan) and the lowest-energy conformation of the selected compounds. **B** 2D representation of the different types of bonds formed between the predicted interactive amino acids of CYP3A4 and the respective selected compounds as visualized by Discovery Studio Visualizer software. The lowest binding energies (LBE) and the predicted inhibition constant (p*K*_*i*_) values for each compound with CYP3A4 are highlighted based on the molecular docking results obtained from AutoDockTools. Chemical structures are displayed according to the color code in panel (**A**)

To further investigate the binding site of the compounds to CYP3A4, we used AutoDock 4.2 as a molecular docking program. We selected only those compounds (1) with the lowest binding energies (LBE) smaller than −8.0 kcal/mol, (2) which are frequently used in the plastic industry and (3) which are commercially available. By applying these criteria, we reduced the number to six selected compounds (from now on referred to as compounds **1** to **6**). Compounds **1** and **2** are plasticizers, compound **3** is a UV stabilizer, and compounds **4**, **5**, and **6** are antioxidants. The CYP3A4-binding candidates showed LBE values between −12.05 and −9.07 kcal/mol and prediction inhibition constants (p*K*_*i*_) between 1.47 and 226.27 nM. The visualization of the molecular docking results displays all compounds in the LBE conformation. In Fig. 2, the interactions between the six candidate compounds and the protein of interest are displayed.

### Cytotoxicity assay

**Fig. 3:**
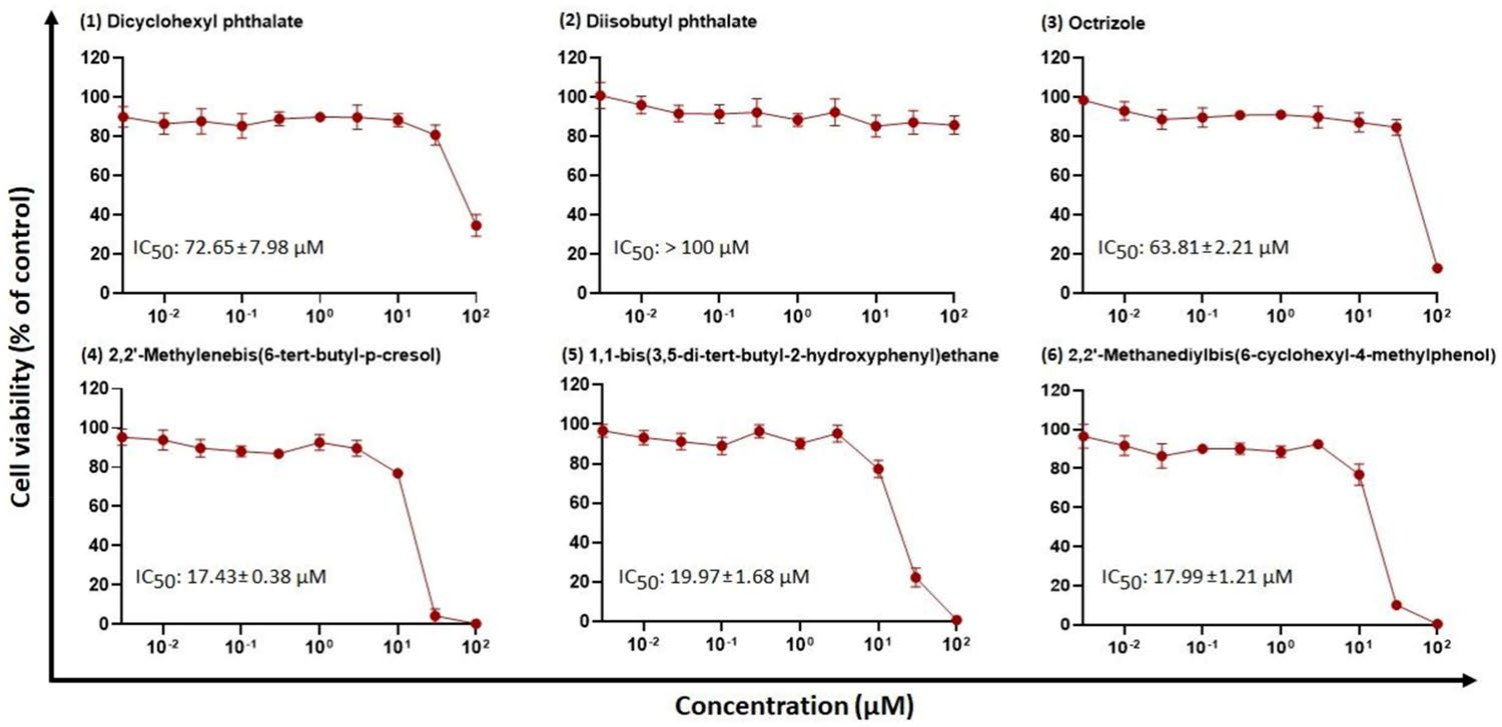
Growth inhibition assay of cytochrome P450 3A4-overexpressing HepG2 cells treated with different concentrations of six selected compounds. The results are represented as mean values ± SD of three independent experiments

We examined the in-silico effect of the candidates on cell viability, using a CYP3A4-overexpressing HepG2 cell line (Fig. 3). Concentrations ranging from 0.003 to 100 μM were tested. The dose–response curves and the IC_50_ values are shown in Fig. 3. The IC_50_ values ranged from 17.43 (± 0.38) μM (compound **4**) to 72.65 (± 7.98) μM (compound **1**).

### RNA-sequencing

We identified differentially expressed genes comparing cells treated with compounds **4**, **5**, and **6** with control cells without treatment (only DMSO). Afterwards, we performed functional enrichment analysis with clusterProfiler (Wu et al. 2021), using the Gene Ontology Biological Process annotation. Then, we generated a visual summary of the affected pathways with GeneTonic software (Marini et al. 2021), further referred to as ‘enriched pathways’.

**Fig. 4:**
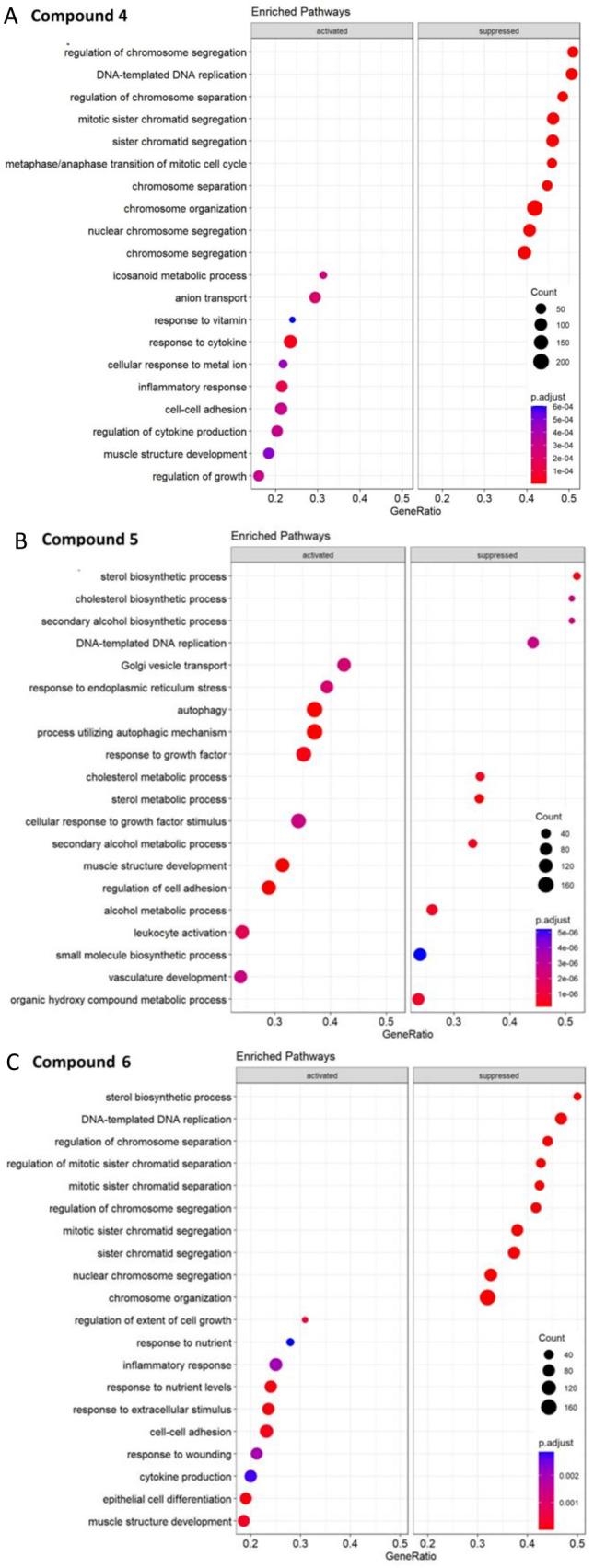
Gene set enrichment analysis showing the top 10 upregulated and the 10 downregulated pathways. **A** Enriched pathways referred to compound **4**. **B** Enriched pathways referred to compound **5**. **C** Enriched pathways referred to compound **6**. Pathways with a positive enrichment score were considered activated and with a negative score as down-regulated. The size of the dots corresponds to the number of genes in the reference gene set. The color of the dots corresponds to the adjusted *p*-value

Figure 4 shows the top 10 downregulated and the top 10 upregulated pathways. Inspecting the enriched pathways maps in more detail showed that there was one pathway commonly regulated by all three compounds, *i.e*., the suppression of ‘DNA-templated DNA replication’. As DNA replication is important for cell division and growth, we also inspected the expression patterns in our data with respect to these two cellular functions. We observed several suppressed mitosis-related pathways that were in common between compounds **4** and **6** (i.e., ‘regulation of chromosome segregation’, ‘regulation of mitotic sister chromatid separation’, ‘nuclear chromosome segregation’, and ‘chromosome organization’). The pathway ‘metaphase/anaphase transition of mitotic cell cycle’ appeared for compound **4** only. By contrast, several cell growth-related pathways were activated, e.g., ‘regulation of growth’ (compound **4**), ‘response to growth factors’ and ‘cellular response to growth factor stimulation’ (compound **5**), and ‘regulation of extent of growth’ (compound **6**). On the other hand, stress and cell death pathways were activated by compound **5** (‘autophagy’, ‘process utilizing autophagic mechanism’, and ‘response to endoplasmic reticulum stress’) and compound **6** (‘response to wounding’). Interestingly, inflammation-related pathways were activated by the compounds: ‘response to cytokines’ (compound **4**), ‘inflammatory response’ (compounds **4** and **6**), and ‘cytokine production’ (compounds **5** and **6**). Furthermore, several pathways associated with metabolic and biosynthetic functions were deregulated. Pathways activated by compound **4** were ‘icosanoid metabolic process’, ‘anion transport’, and ‘response to metal ions’. ‘Golgi vesicle transport’ was activated by compound **5**. ‘Response to nutrients’ and ‘response to nutrients levels’ were activated by compound **6**. Treatment with compound **4** suppressed ‘response to vitamin’, and with compound **5** ‘cholesterol metabolic process’, ‘cholesterol biosynthetic process’, ‘secondary alcohol biosynthetic process’, ‘sterol metabolic process’, ‘alcohol metabolic process’, ‘small molecule metabolic process’, and ‘organic hydroxy compound metabolic process’. Finally, ‘sterol biosynthetic process’ was suppressed by compound **6**. By using the GeneTonic software, we generated heat maps of certain pathways that were in common among the three compounds. The heat maps of the three compounds vs ctrl (DMSO) regarding the ‘DNA-templated DNA replication’ pathway are shown in Supplementary Fig. 1A. There are numerous genes being targeted by the investigated compounds. Therefore, we decided to further examine which of these genes are mutually affected by the three compounds. There were 78 genes targeted by compound **4**, 68 for compound **5**, and 72 for compound **6**, respectively. The Venn diagram in Supplementary Fig. 1B shows the genes in common among the various compounds. We observed that 49 genes were commonly affected by all three compounds, 6 genes by compound **4** and **5**, 13 by compounds **4** and **6**, and 5 genes by compounds **5** and **6**. In contrast, 10 genes for compound **4**, 8 genes for compound **5**, and 5 genes for compound **6** were influenced by only one compound. Furthermore, we generated another heat map showing the log2 fold-change of each gene in common for all three compounds (Supplementary Fig. 1C). Some genes were more strongly down-regulated by all of them such as *POLA2* (DNA Polymerase α2 Accessory Subunit) or *TICRR* (TOPBP1 Interacting Checkpoint and Replication Regulator), while other genes showed different behavior in relation to the compound. Indeed, genes such as *E2F7* (E2F Transcription Factor 7) or *GINS4* (GINS Complex Subunit 4) are more affected by compounds **4** and **5** than compound **6**. However, compound **6** has a higher impact on genes such as *FEN1* (Flap Structure-Specific Endonuclease 1) or *RFWD3* (Ring Finger and WD Repeat Domain 3). Heat maps on the effects of compounds **4** and **6** on the ‘nuclear chromosome segregation’ pathway are shown in Supplementary Fig. 2A. The Venn diagram in Supplementary Fig. 2B shows that there were 103 genes affected by compound **4** and 83 by compound **6**. Of them, 73 genes were in common. The heat map in Supplementary Fig. 3C depicts that compound **4** generally had a greater influence on genes related to the ‘nuclear chromosome segregation’ pathway than compound **6**. The two heat maps related to ‘inflammatory response’ pathway that was over-expressed upon treatment with compounds **4** and **6** are shown in Supplementary Fig. 3A. Numerous genes were involved (76 for compound **4** and 90 for compound **6**) as shown in Supplementary Fig. 3B. Of them, 63 were in common. Many of these genes showed a marked over-expression after treatment with both compounds such as *SERPIN1* (Serpin Family E Member 1), *NUPR1* (Nuclear Protein 1) or *AXL* (AXL Receptor Tyrosine Kinase) (Supplementary Fig. 3C).

### Cell cycle analysis

**Fig. 5:**
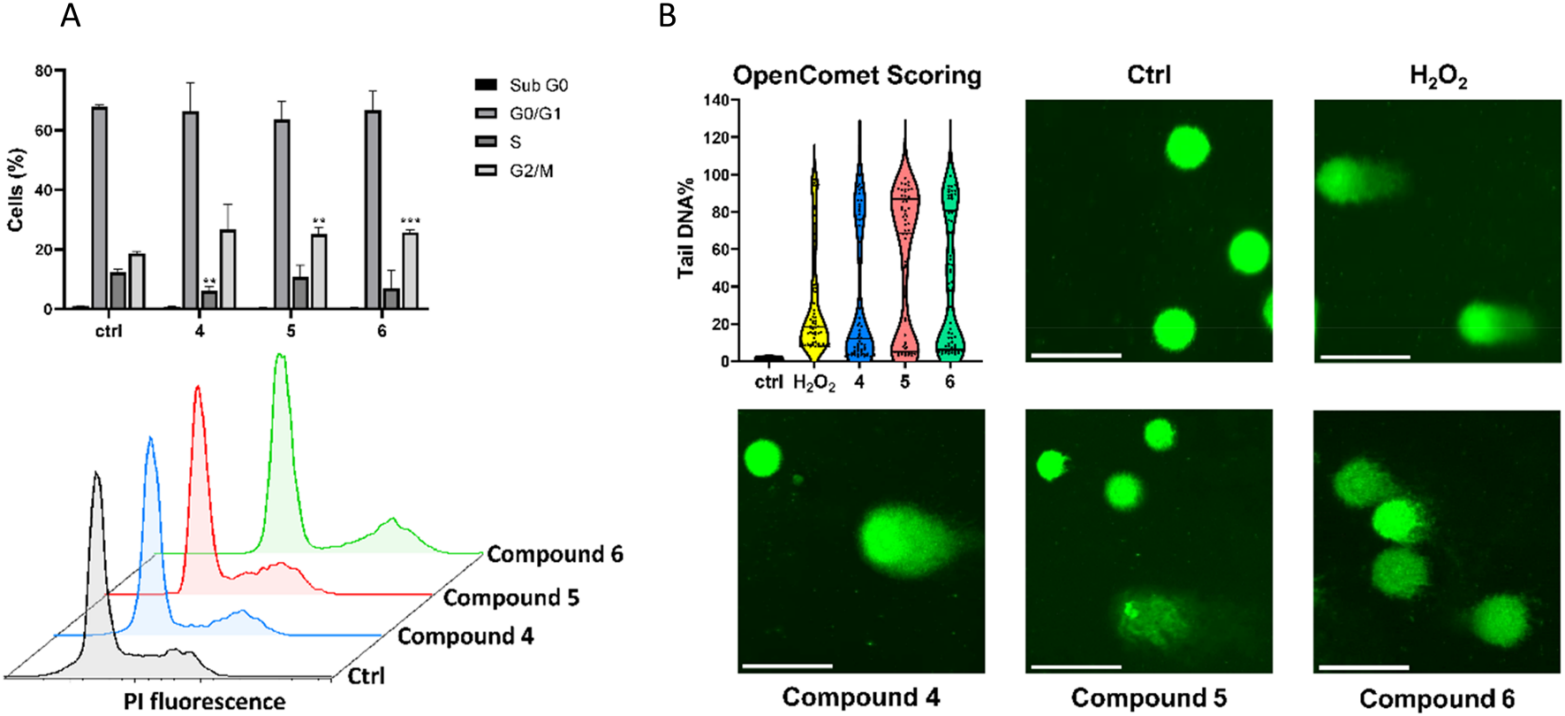
**A** Cell cycle analysis. Flow cytometric cell cycle analysis in CYP3A4-overexpressing HepG2 cells treated with IC_50_ concentration of compound **4**, **5**, and **6**. The data are represented as mean values ± SD of three independent experiments. (**p < 0.01, ***p < 0.001, compared to ctrl (DMSO) cells). **B** Detection of DNA damage by alkaline comet assay in CYP3A4-overexpressing HepG2 cells. Cells incubated with IC_50_ concentration of compound **4**, **5**, and **6** for 24 h and 50 μM of H_2_O_2_ (positive control) for 1 h. The parameter tail DNA% was measured from 60 randomly selected cells shown in the violin plot. Scalebar: 100 μm

The results of the flow cytometric cell cycle analysis are shown in Fig. 5A. The treatment with the three selected compounds disclosed an arrest in G2/M phase at the IC_50_ concentration (26.70 ± 8.40% for compound **4**, 25.13 ± 2.25% for compound **5** and 25.77 ± 0.95% for compound **6**, respectively) compared to the control cells (18.70 ± 0.69%). Moreover, the analysis of compound **4** showed a significantly reduced percentage of S-phase cells population (6.26 ± 1.31%) in contrast to the control (12.47 ± 0.83%). On the other hand, compounds **5** and **6** displayed only a slightly decreased percentage of S phase (10.88 ± 3.84 and 7.06 ± 5.94%, respectively). No significant percentage change was detected in the sub-G0 and G0/G1 phases.

### Single-cell gel electrophoresis

We examined DNA damage at the level of individual cells by means of the alkaline comet assay. During electrophoresis, damaged DNA or denatured cleaved DNA fragments migrate from intact cells, creating a “comet tail” under the microscope. All three compounds induced comet tails in CYP3A4-overexpressing cells upon treatment with IC_50_ concentrations (Fig. 5B). Compared to undamaged control cells, there was an increase in comet tails induced by all three compounds, suggesting that DNA was indeed damaged. H_2_O_2_, as a positive control, also produced clearly visible comet tails. Analysis of the tails of 60 randomly selected cells, revealed that the three compounds trigger marked DNA damage.

### Predicted metabolites

Finally, we were interested in whether the metabolization of these compounds by CYP3A4 might influence their binding affinities. Using the software smartCYP (Rydberg et al. 2010a, b) we predicted the possible metabolites of compounds **4**, **5**, and **6**. In Supplementary Fig. 4A, the possible sites of hydroxylation by CYP3A4 are shown. In Supplementary Fig. 4B, the lowest binding energies (LBE) of the metabolites of the various compounds in different combinations are highlighted in comparison with the unmodified molecules. For compound **4**, the values ranged from −10.58 kcal/mol (referring to hydroxylation of C.13) to −11.63 kcal/mol (referring to hydroxylation of C.13, C.1, C.7). For compound **5**, the highest value was −13.02 kcal/mol (referring to the modifications on C.2 and C.6), while the lowest value was found with the modification of C.21 with a value of −14.32 kcal/mol. Finally, the highest value was −12.28 kcal/mol (modification of C.8) and −13.70 kcal/mol for the lowest value (modification of C.15 and C.8) for compound **6**. In general, we noted an increase in the binding affinities to CYP3A4 for all three compounds after metabolization.

## Discussion

The possible influence of plastic, microplastic, and related compounds on our health is rather elusive to date. Although the fundamental role of CYPs on cellular homeostasis, the metabolism of toxic substances (Manikandan and Nagini 2017), and the interaction with environmental pollutants such as pesticides or chemicals (Hodgson and Rose 2007) has been previously investigated, studies on plastic-related compounds remain scarce. Considering the increasing presence of plastic in our daily life (Ellen MacArthur Foundation and World Economic Forum 2016), it is not surprising that an intensified use in the future is expected. Studies have shown the emission of plastic-related compounds into the environment (Cooper et al. 2011; Kwan and Takada 2019), emphasizing the necessity to investigate possible toxic effects for humans.

In the present study, we investigated the possibly toxic interactions of six compounds that are all involved in plastic production, with CYP3A4 as one of the most important CYP enzymes. We first screened a library of plastic-related chemicals and reanalyzed them by molecular docking followed by cytotoxicity analyses. We selected three compounds with good binding affinities to CYP3A4 and low IC_50_ values (< 20 µM). Once the cytotoxic action of the three compounds was demonstrated, we continued our studies at the transcriptomic level using RNA sequencing. Several studies have shown that the interaction between environmental pollutants (e.g., agricultural pollutants or persistent organic pollutants) and CYPs (Saintot et al. 2004; Lagunas-Rangel et al. 2022) triggered altered regulation at the gene transcript level. Comparable effects were noted with hepatotoxic compounds such as pyrrolizidine alkaloids (Abdelfatah et al. 2022) or carcinogenic polycyclic aromatic hydrocarbons (Hodgson and Rose 2007), enabling the study of xenobiotic compounds at the transcriptomic level. Using RNA-sequencing data, we identified common pathways influenced by three selected plastic-related compounds. In particular, several pathways involved in mitosis (such as ‘regulation of chromosome segregation’ or ‘chromosome organization’) for compounds **4** and **6** were suppressed, while the ‘DNA-template DNA replication’ pathway was downregulated for all three compounds.

Using the flow cytometer, we observed that all three compounds induce a cell cycle arrest in the G2/M phase, combined with a decrease in the S phase. It is well known that the G2/M phase arrest represents a key cellular response to DNA damage, preventing the transmission of impaired DNA for mitosis without repair of DNA lesions (Schönthal 2004). Therefore, to assess the integrity of DNA in CYP3A4-overexpressing HepG2 cells after treatment with plastic-related compounds, we performed the alkaline comet assay. In line with the arrest in G2/M phase previously detected with the cell cycle analysis, all three compounds showed severe DNA damage. Thus, these findings are consistent with the pathways identified through RNA-sequencing analysis.

There are several studies in which plastic-related compounds (Xu et al. 2002; Erkekoglu et al. 2010; Erkekoglu and Kocer-Gumusel 2014; Chen et al. 2021; Kumari et al. 2021) or environmental pollutants (Somers et al. 2002; Gillings et al. 2018; Rider and Carlsten 2019) caused cytotoxicity, DNA damage or blockage of cells in the G2/M phase. In this context, the reduced capacity for mitosis and DNA replication impairs the regenerative capacity of the liver which is known to be very efficient under normal conditions (Fausto et al. 2006).

By contrast, those pathways leading to cell growth (such as ‘regulation of growth’, ‘response to growth factors’ or ‘regulation of extent of growth’) were activated by all three compounds. This behavior could represent a compensatory mechanism used by liver cells to counteract the inhibition of mitosis. However, it is crucial to note that the activation of growth pathways may potentially contribute to malignant progression (DeBerardinis et al. 2008; Heiden Vander et al. 2011). Indeed, strong growth signaling may override the suppression of mitosis, but this could lead to improperly executed cell divisions and the promotion of chromosomal abnormalities. Such chromosomal abnormalities are known to be associated with cancer development and progression (Solomon et al. 1991). Alternatively, suppression of the G2/M phase may lead to cell death (Duan et al. 2016; Wang et al. 2020). In fact, uniquely for compound **5**, showing both strong DNA damage and G2/M phase arrest, an activation of autophagy pathways (such as ‘autophagy’ and ‘process utilizing autophagic mechanism’) was noted. Furthermore, the activation of inflammation-related pathways by all three compounds (such as ‘inflammatory response’ and ‘cytokine production’) suggests that plastic-related compounds may trigger inflammatory responses in the liver. The induction of inflammatory processes by plastics and microplastics (Jin et al. 2018; Yang et al. 2022) but especially by plastic-related compounds has been reported (Murata and Kang 2018). However, it can have damaging effects in the liver (Sorci and Loiseau 2022), including the initiation and progression of liver diseases such as fibrosis and potentially cirrhosis (Horvatits et al. 2022). Therefore, the inflammatory response provoked by plastic-related compounds further contributes to their overall toxic effect on hepatocytes.

In addition to these harmful effects, disturbances were also observed in numerous liver functions, including ‘sterol biosynthetic process’, ‘Golgi vesicle transport’, or ‘cellular response to metal ion’. These dysregulations further emphasize the multifunctional nature of the toxic effects induced by plastic-related compounds. Disruptions in the 'sterol biosynthetic process', where cholesterol represents the most important product, were shown for compounds **5** and **6** and may not only have profound implications on cell homeostasis (Subczynski et al. 2017) but also on the entire organism (Schade et al. 2020). Interference with the metal ion response (compound **4**) also causes various problems. Indeed, metallothioneins in this pathway play a key role in metal homeostasis as well as protection from heavy metal toxicity, DNA damage and oxidative stress (Si and Lang 2018). Their disruption would exacerbate liver toxicity. Finally, compound **5**, showed significant overexpression of ‘Golgi vesicle transport’ pathway, which assumes an important role in cellular homeostasis by regulating protein, lipid modification and sorting (Liu et al. 2021). Its dysregulation leads to various disorders in different organs, not only in the liver (Liu et al. 2021). Furthermore, metabolites of the main compounds can also have toxic effects (Guengerich et al. 1985). In this research, we observed an increased binding affinity of the metabolites to CYP3A4 leading us to the assumption that they may also exert similar effects as the main molecules. Nowadays, we can encounter many types of plastics and microplastics through different intake routes (Cox et al. 2019). Studies have been conducted regarding the translocation of microplastics and plastic-related compounds across the gastrointestinal epithelial barrier (Wright and Kelly 2017), subsequently gaining access to the circulatory system (Leslie et al. 2022). Then, these particles are transported to the liver through the portal vein, where they interact with hepatocytes expressing the enzyme CYP3A4. Plastic-related compounds have the capacity to accumulate in various organs and compartments, including the liver and adipose tissue (Mes et al. 1974; Bell 1982; Ganning and Dallner 1984). Consequently, it is plausible to hypothesize that sustained daily exposure (Koelmans et al. 2019; Sánchez 2022) to these compounds and their metabolites, coupled with progressive accumulation, may result in the gradual escalation of local concentrations in the body over an extended period. Such protracted exposure and accumulation may potentially lead to chronic toxicity (Pereira et al. 2006; Horvatits et al. 2022; Yin et al. 2022). In this study, we were able to analyze the effect of three selected plastic-related compounds and determine the commonly affected pathways. Within these pathways we could observe a high number of genes which were targeted by at least two of the selected compounds. Considering these findings, an amplified toxicity may occur when exposed to several plastic related compounds because they may target common genes.

## Conclusions

Our study highlights the complex and multifaceted nature of the effects of plastic-related compounds on liver cell functions. Selected compounds (**4**, **5**, and **6**) interfering with critical cellular processes such as mitosis and DNA replication, therefore, lead to cytotoxicity and DNA damage. The observed activation of growth pathways which can induce a subsequent potential of malignant progression may indicate the intricate balance between cellular compensation mechanisms and the risk of carcinogenesis. Furthermore, the induction of inflammatory responses in the liver, as well as the disruption of other metabolic pathways related to molecules essential to the body, underlines the broader implications for human health. Further in-vivo research is needed to investigate the multitude of observed effects, as plastic-related compounds do not act in a monocausal but multifunctional manner. Knowing that the devastating plastic pollution crisis will inevitably gain in severity, in our opinion, the scientific community must begin to not only promote scientific research on this topic but also improve communication with the general public. Thus far, plastic pollution has been mainly evaluated as an environmental issue, but its relevance for human health must be further emphasized. In this context, our findings shed new light on the possible toxicity of plastic-related compounds.

### Supplementary Information

Below is the link to the electronic supplementary material.

Supplementary file1 (DOCX 2170 KB)

## Data Availability

All sequencing datasets generated for this study have been deposited in the Gene Expression Om-nibus (GEO) under accession number GSE237739.

## Abbreviations

CYPs: Cytochrome P450 monooxygenases
Ctrl: Control
DMSO: Dimethyl sulfoxide
PBS: Phosphate-buffer saline
H_2_O_2_: Hydrogen peroxide
PI: Propidium iodide
