# Supplementary material for: Impact of plastic-related compounds on the gene expression signature of HepG2 cells transfected with CYP3A4
Source: Arch Toxicol. 2023 Dec 30;98(2):525–36. doi: 10.1007/s00204-023-03648-4 (PMC10794370; doi:10.1007/s00204-023-03648-4)
Supplement: Supplementary file 1 — Supplementary file1 (DOCX 2170 KB) [file 204_2023_3648_MOESM1_ESM.docx]

**Supplementary Material**

**Impact of plastic-related compounds on the gene expression signature of HepG2 cells transfected with CYP3A4**

**Matteo Rosellini^1^, Ejlal A. Omer^1†^, Alicia Schulze^2†^, Nadeen T. Ali^1^, Joelle C. Boulos^1^, Federico Marini^2,3^, Jan-Heiner Küpper^4^, Thomas Efferth^1*^**

^1^ Department of Pharmaceutical Biology, Institute of Pharmaceutical and Biomedical Sciences, Johannes Gutenberg University, Staudinger Weg 5, 55128 Mainz, Germany.

^2^ Institute of Medical Biostatistics, Epidemiology and Informatics (IMBEI), Medical Center of the Johannes Gutenberg University, 55122 Mainz, Germany.

^3^ Research Center for Immunotherapy (FZI), Langenbeckstraße 1, 55131 Mainz.

^4^ Institute of Biotechnology, Brandenburg University of Technology Cottbus-Senftenberg, 03046 Senftenberg, Germany.

†These authors contributed equally to this work and share second authorship.

*** Correspondence:**Corresponding Author
[efferth@uni-mainz.de](mailto:efferth@uni-mainz.de)


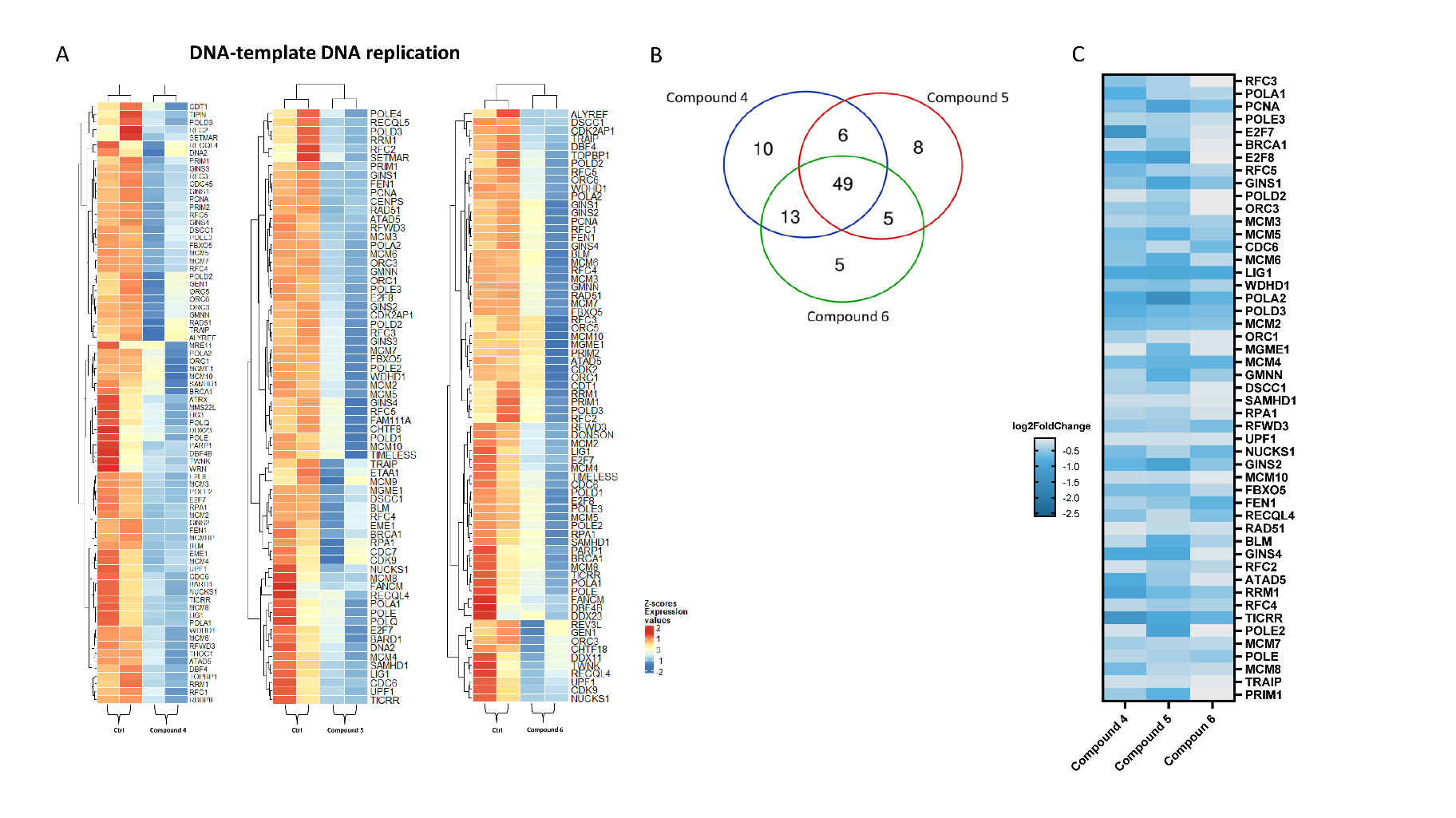


**Supplementary Figure 1.** (A) Heat maps of differentially expressed genes related to ‘DNA-template DNA replication’ pathway upon treatment with the three selected compounds. The samples selected for comparison always refer to the compound of interest vs. Ctrl (DMSO). Color-coded standardized z-scores for the expression values after variance stabilizing transformation was used to simplify comparison across samples. (B) Venn diagram of the genes in common amongst the three selected compounds. (C) Heat map showing log2 fold-changes of significantly down-regulated genes in common among the three compounds.


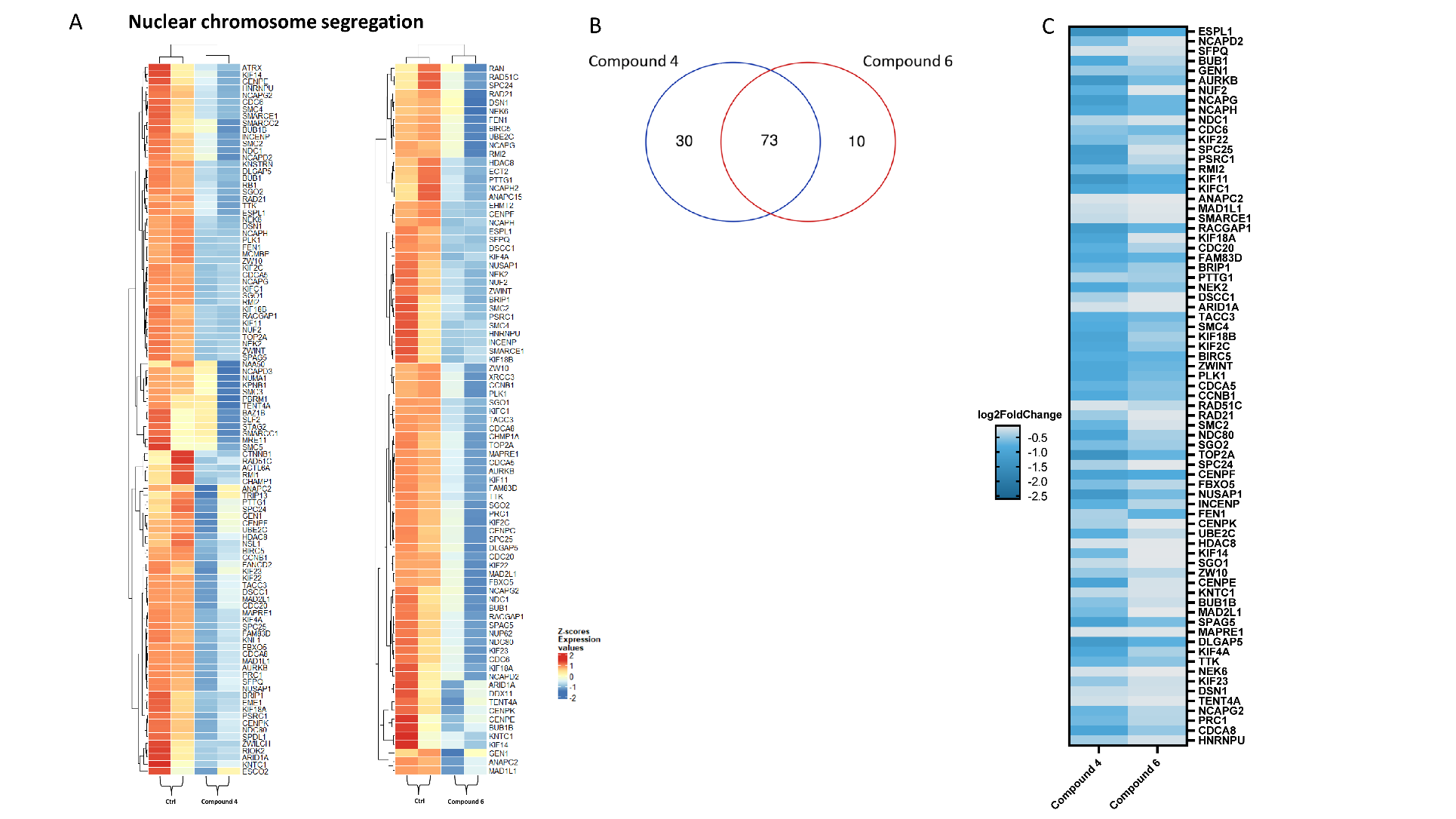


**Supplementary Figure 2.** (A) Heat maps of differentially expressed genes related to ‘nuclear chromosome segregation’ pathway upon treatment of compounds 4 and 6. The samples selected for comparison always refer to the compound of interest vs. Ctrl (DMSO). Color-coded standardized z-scores for the expression values after variance stabilizing transformation was used to simplify comparison across samples. (B) Venn diagram of the genes in common amongst the two selected compounds. (C) Heat map showing log2 fold-changes of significantly down-regulated genes in common among the two compounds.


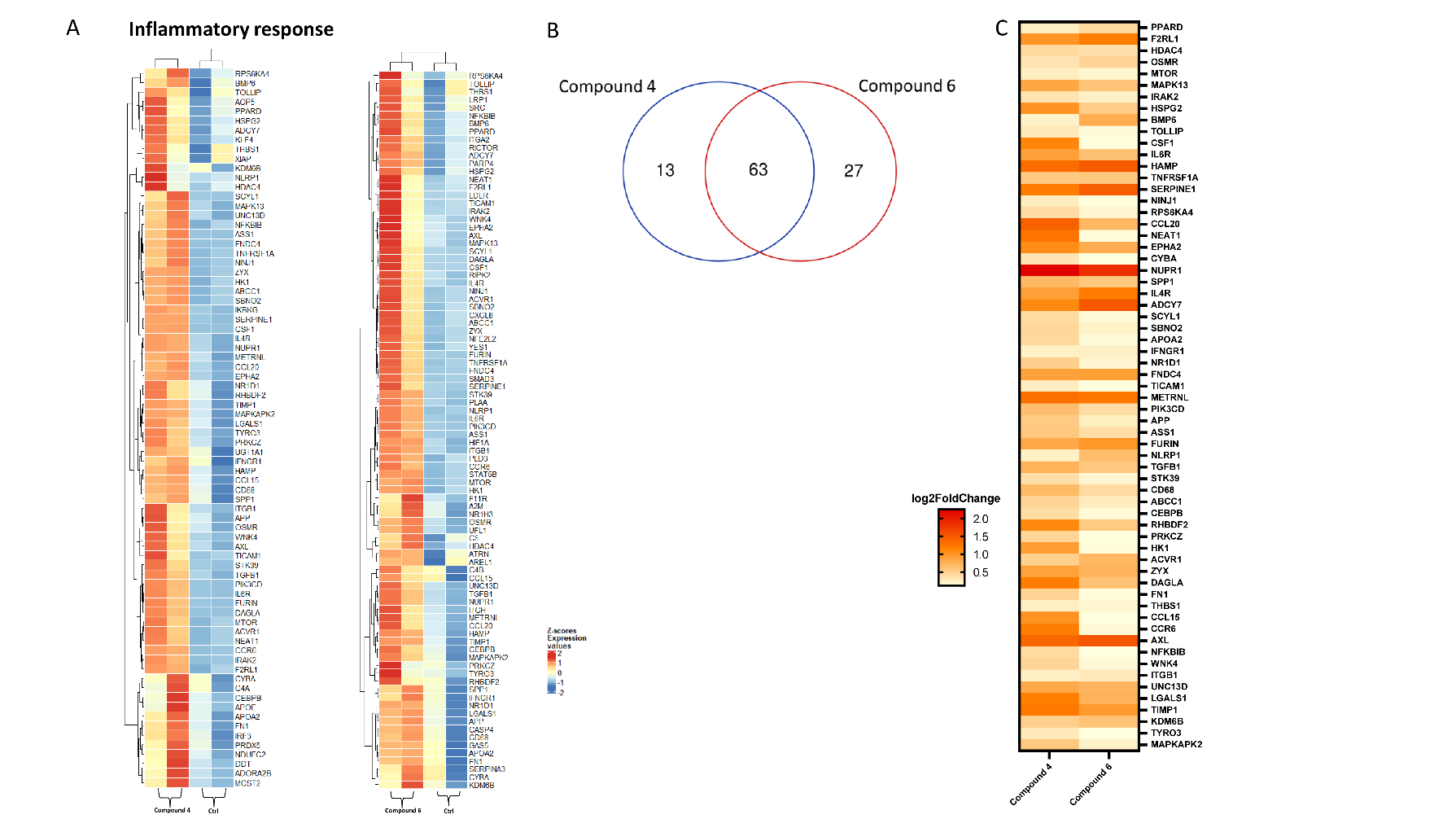


**Supplementary Figure 3.** (A) Heat maps differentially expressed genes related to ‘inflammatory response’ pathway upon treatment of compounds 4 and 6. The samples selected for comparison always refer to the com-pound of interest vs. DMSO. Color-coded standardized z-scores for the expression values after variance stabilizing transformation was used to simplify comparison across samples. (B) Venn diagram of the genes in common amongst the two selected compounds. (C) Heat map showing log2 fold-changes of significantly up-regulated genes in common among the two compounds.


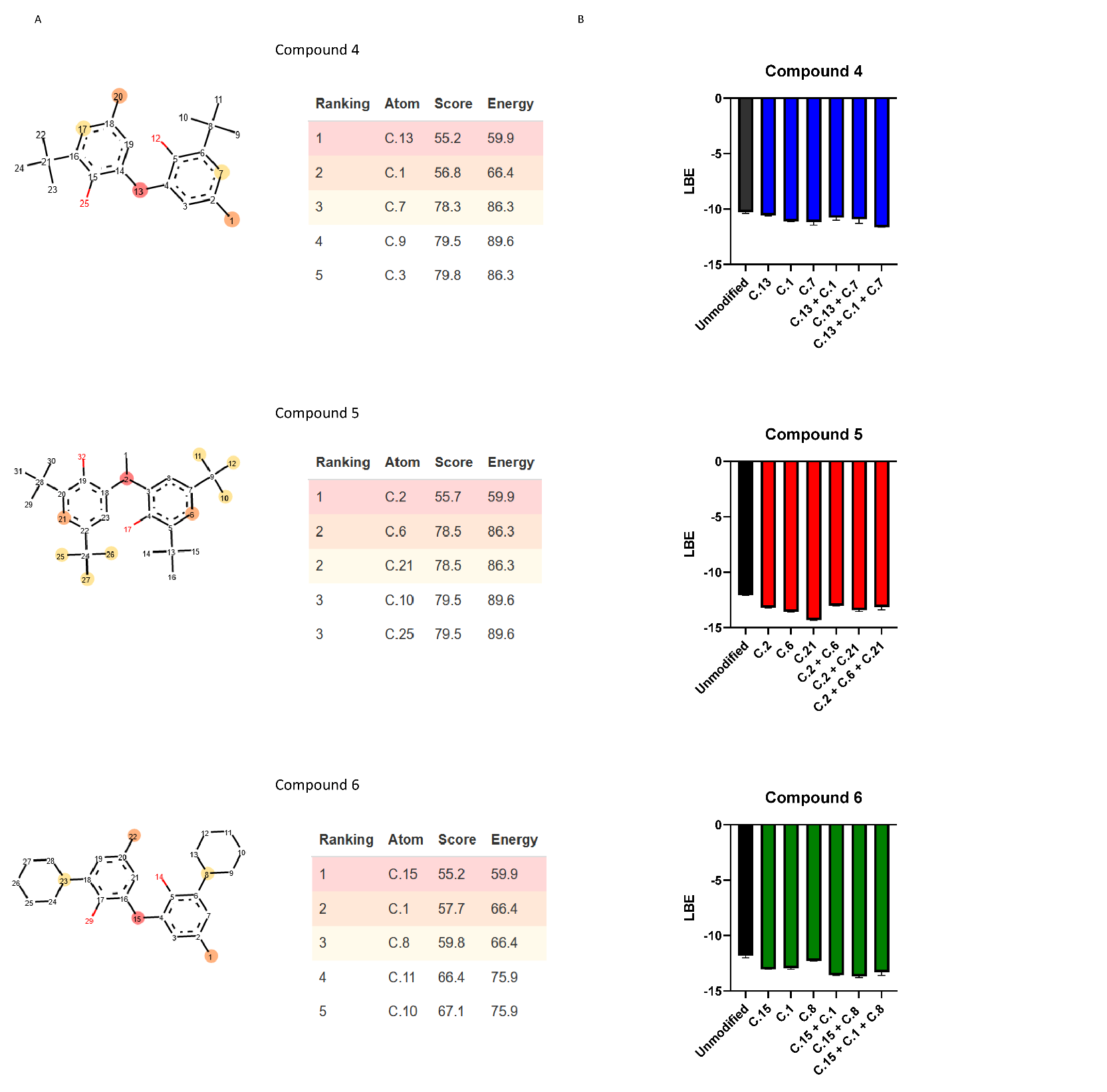


**Supplementary Figure 4.** Predicted sites of metabolism and lowest binding energy (LBE) for the selected compounds. (A) Possible sites of hydroxylation by CYP3A4 for the three selected compounds with the top5 ranking position. (B) Lowest binding energies (LBE) of the metabolites of the various compounds in different combinations in comparison with the unmodified molecule.
